# *In Vitro* Study of *Taenia solium* Postoncospheral Form

**DOI:** 10.1371/journal.pntd.0004396

**Published:** 2016-02-10

**Authors:** Nancy Chile, Taryn Clark, Yanina Arana, Ynes R. Ortega, Sandra Palma, Alan Mejia, Noelia Angulo, Jon C. Kosek, Margaret Kosek, Luis A. Gomez-Puerta, Hector H. Garcia, Cesar M. Gavidia, Robert H. Gilman, Manuela Verastegui, Cysticercosis Working Group in Peru

**Affiliations:** 1 Department of Cellular and Molecular Sciences, Universidad Peruana Cayetano Heredia, Lima, Peru; 2 Section of Emergency Medicine, Department of Medicine, Louisiana State University Health Sciences Center, New Orleans, Louisiana, United States of America; 3 Center for Food Safety and Department of Food Science and Technology, University of Georgia, Griffin, Georgia, United States of America; 4 Department of Pathology, School of Medicine, Stanford University, Stanford, California, United States of America; 5 Department of International Health, Bloomberg School of Hygiene and Public Health, Johns Hopkins University, Baltimore, Maryland, United States of America; 6 School of Veterinary Medicine, Universidad Nacional Mayor de San Marcos, Lima, Peru; 7 Asociación Benéfica PRISMA, San Miguel, Lima, Peru; University of Würzburg, GERMANY

## Abstract

**Background:**

The transitional period between the oncosphere and the cysticercus of *Taenia solium* is the postoncospheral (PO) form, which has not yet been completely characterized. The aim of this work was to standardize a method to obtain *T*. *solium* PO forms by *in vitro* cultivation. We studied the morphology of the PO form and compared the expression of antigenic proteins among the PO form, oncosphere, and cysticerci stages.

**Methodology/Principal Findings:**

*T*. *solium* activated oncospheres were co-cultured with ten cell lines to obtain PO forms, which we studied at three stages of development–days 15, 30, and 60. A high percentage (32%) of PO forms was obtained using HCT-8 cells in comparison to the other cell lines. The morphology was observed by bright field, scanning, and transmission electron microscopy. Morphology of the PO form changed over time, with the six hooks commonly seen in the oncosphere stage disappearing in the PO forms, and vesicles and microtriches observed in the tegument. The PO forms grew as they aged, reaching a diameter of 2.5 mm at 60 days of culture. 15–30 day PO forms developed into mature cysticerci when inoculated into rats. Antigenic proteins expressed in the PO forms are also expressed by the oncosphere and cysticerci stages, with more cysticerci antigenic proteins expressed as the PO forms ages.

**Conclusions/Significance:**

This is the first report of an *in vitro* production method of *T*. *solium* PO forms. The changes observed in protein expression may be useful in identifying new targets for vaccine development. *In vitro* culture of PO form will aid in understanding the host-parasite relationship, since the structural changes of the developing PO forms may reflect the parasite’s immunoprotective mechanisms. A wider application of this method could significantly reduce the use of animals, and thus the costs and time required for further experimental investigations.

## Author Summary

Neurocysticercosis is caused by *T*. *solium*, which is a neglected disease. The postoncospheral (PO) form is an intermediate form between the oncosphere, which is the larva, and the fully developed cysticercus, which is a cyst with a scolex. The morphology, development, and protein and antigen expression of the PO form have not previously been characterized. Here, we report the novel *in vitro* cultivation of *T*. *solium* PO forms and characterize the morphology, development, and expression of antigenic proteins. This new method will allow for better study of this transitional form, which is very difficult to study in the intermediate host. With the increased availability of secreted proteins and antigens, *in vitro* cultivation will help improve diagnostic assays and provide new targets for vaccine development to block transmission.

## Introduction

Human and porcine cysticercosis is a disease caused by the larval cestode *Taenia solium* which forms cysts in the muscles or the central nervous system of its intermediate host. Both humans and pigs can acquire cysticercosis through fecal-oral transmission by ingesting *T*. *solium* eggs released by the adult tapeworm, which lives exclusively in the small intestines of humans. This parasitic disease is a major public health problem in developing countries where pigs are raised as a food source and causes great economic loss to farmers. It is strongly correlated with poor sanitary conditions and deficient sanitation infrastructure in regions where pig rearing is common [1], [2], [3].

When cysticercosis involves the central nervous system in humans, it is called neurocysticercosis (NCC). NCC is common throughout Latin America, Sub-Saharan Africa, most of Asia, and parts of Oceania. Human NCC is believed to be the leading cause of acquired epilepsy worldwide [4], [5].

The eggs of *T*. *solium* contain a six-hooked larva (hexacanth) called the oncosphere [6]. When the egg hatches, this oncosphere is released into the intestine. Gastric fluid and intestinal fluid might work together to dissolves the cementing material of the embryophore shell blocks and releases the unactivated oncosphere. The oncosphere is then stimulated by the intestinal fluid to activate and to tear open the enclosing oncospheral membrane. This activated oncosphere can penetrate the intestinal wall and reach the target tissues (usually muscle or the central nervous system) where it transforms into a cysticercus. This is the larval stage of the parasite that consists of a fluid-filled sac containing an invaginated scolex [7]. As this happens, the parasite produces a variety of molecules, which modulate the host immune response, in order to evade parasite destruction [7].

There is a postoncospheral (PO) form during the development of the cysticercus. The PO form is an intermediate stage between the oncosphere and the fully developed cysticercus in tissue [7]. This form is also called the early stage of metacestode [8]. In other cestodes, the PO form has been obtained *in vitro* by co-culture of oncospheres with a monolayer of mammalian feeder cells (*Taenia ovis* and *Taenia saginata*) [9], [10] or without feeder cells (*Echinococcus granulosus*, *Taenia hydatigena*, *Taenia ovis*, *Taenia pisiformis*, *Taenia serialis*, *Taenia saginata* and *Taenia taeniaeformis*) [11], [12].

Studies of the *T*. *solium* PO forms have only been done *in vivo* using pigs, focusing on the morphology and distribution of the PO forms [8], [13]. *In vitro* cultivation of *T*. *solium* oncospheres could allow the identification of parasite-related molecules and simplify the availability of high quantities of antigen, specifically proteins and antigens expressed during the early stages of the cyst formation, which are currently very difficult to study in the intermediate host. *In vitro* studies could also help improve diagnostic assays, providing new targets for the development of vaccines blocking transmission, and studying protein expressions that may explain the mechanisms of evasion from the host immune response during the development of cysticerci. Therefore, our study's aim was to evaluate the *in vitro* culture of *T*. *solium* larval stage and to describe the morphological characteristics and protein expressions of the postoncospheral development.

## Methods

### Cell culture preparation

In order to determine which cell line was the most successful in producing PO forms, we evaluated different cell lines: human intestinal cells (HuTu-80, INT-407, HCT-8, HT-29 and CaCo-2 cells), human neuroblastoma cells (SH-SY5Y), human choriocarcinoma cells (BeWo), rat myocardium cells (H9C2), human lung cells (MRC-5), Chinese hamster ovary cells (CHO-K1), African green monkey kidney cells (VERO), and Rhesus monkey kidney cells (LLC-MK2). All of these cell lines were obtained from American Tissue Culture Collection (ATCC, Manassas, VA).

Cells were incubated at 37°C in 5% CO_2_ and grown in specific media as recommended by ATCC (EMEM media for Hutu-80, INT-407, HT-29, CaCo-2 and MRC-5 cells; RPMI for HCT-8, VERO, LLC-MK2; EMEM + F12 media for SH-SY5Y; DMEM media for H9c2; F12K media for BeWo; and HAM-F12 for CHO-K1). All media were supplemented with 10% fetal bovine serum and changed every 2 days. Once cell confluency was obtained, cells were harvested using trypsin-EDTA (Sigma Chemical Co). Cells were placed into 24-well plates (1x10^5^ cells per well), and the maturation assay described below was performed when cells formed a monolayer.

### *Taenia solium* oncosphere preparation

Tapeworms were collected after medical treatment of newly diagnosed patients as described by Jeri et al [14]. Hatching of eggs and oncosphere activation were performed as described by Verastegui et al [15]. Briefly, the eggs were obtained from gravid proglotids of adult tapeworms by gentle homogenization in a 2.5% potassium dichromate solution (Sigma, St. Louis, Missouri). Eggs were then washed three times in distilled water (with centrifugation steps to collect the eggs between washes of 2500 x g per 5 min). The eggs were hatched and oncospheres were released with a solution of 0.75% sodium hypochlorite (Mallinckrodt Baker, Inc, Phillipsburg, NJ) in water for 10 minutes at 4°C. Oncospheres were then washed three times in RPMI medium (Sigma, St. Louis, Missouri), and activated by incubation at 37°C for 45 minutes with artificial intestinal fluid (1 g pancreatin [Sigma Chemical Co., St. Louis, MO], 200 mg Na_2_CO_3_, and 1 ml of real pig bile, with enough RPMI 1640 medium [pH 8.04] to make 100mL). After activation, the oncospheres were washed three times with RPMI medium and counted using a Neubauer chamber.

### *In vitro* maturation assay to obtain PO form

We performed three experiments to define the best conditions to obtain PO forms. A parasite was considered to have developed to PO form if we observed the following characteristics: increased number of cells, increased size, loss of hooks, formation of inner cavity with no scolex, and change of morphology.

**The first experiment** was aimed at identifying the best cell line to obtain PO forms. Ten thousand activated oncospheres were placed in each well of 24-well plates containing one of the different confluent cell lines listed above; they were incubated in 5% CO_2_ at 37°C, and the cell medium was changed every 3 days for a period of two weeks. This experiment was repeated three times.

The free-floating PO forms were collected and rinsed three times with sterile PBS buffer. Washes consisted of transferring the PO forms into a 1.5 mL tube, allowing it to settle for 5 minutes, then the supernatant was removed and 1 mL of sterile PBS was added before the process was repeated. Finally, the PO forms were resuspended in 1 mL of PBS, mixed gently, and counted using an inverted microscope.

**The second experiment** aimed to evaluate if the oncosphere can develop to PO form in the absence of a cell monolayer. Based on the results of the first experiment, activated oncospheres (n = 10,000) were cultured for two weeks in: A) HCT-8 cell monolayer (positive control), B) culture media alone, C) supernatant from HCT-8 cell monolayer and D) supernatants from HCT-8 cell monolayer that had been incubated with 2-week old PO forms.

**The third experiment** was done to evaluate if the oncosphere could develop into PO form in the absence of direct contact with the selected feeder cell (HCT-8). We co-cultured the feeder cells and activated oncospheres (n = 10,000) in two types of transwell systems; one with a collagen-coated membrane (Transwell-PTFE/COL) and the other one with a polyester membrane (Transwell-PET). The activated oncospheres were added to the top of each transwell insert (3.0 μm pore size; 24-well). One milliliter of medium was placed in the bottom well, which contains the HCT-8 monolayer cells. Two types of controls were included in this experiment; one with activated oncospheres in a transwell system (collagen or polyester membrane) in the absence of monolayer cells, and the second one with activated oncospheres in direct contact with the monolayer cells. The 24-well plate was incubated for two weeks in 5% CO_2_ at 37° and the medium in the bottom well was replaced every three days.

### *In vitro* study of the PO form morphology

The HCT-8 intestinal cell line was selected to study the morphological changes of the parasite because it yielded the highest percentage of PO forms. Ten thousand activated oncospheres were cultured in confluent HCT-8 cell monolayer and cell medium was changed every three days. The free-floating PO forms were collected and rinsed twice with fresh medium, then transferred to another well containing confluent HCT-8 cell monolayers. This process was repeated every three days for up to two months to allow the PO form to continue development. Cultures were inspected daily using an inverted microscope (Leitz labovert FS). PO forms were collected at 15, 30, and 60 days of incubation. After 2 months of incubation, the PO forms began to die off.

The PO forms collected were divided into 3 aliquots. The first aliquot was dried on a slide coated with poly-L-Lysine 0.1% solution (Sigma, St. Louis, Missouri) followed by fixation with methanol-acetone (1:1) for 10 min at -20°C, and the slides were then washed three times with PBS, mounted with Prolong gold antifade reagent with DAPI (Life Technologies, Carlsbad, CA), and examined by UV microscopy (Leica DM 500). The second aliquot was fixed with 2% of glutaraldehyde (Grade I: 70% solution) in 1% sucrose in PBS to be examined by scanning electron microscopy (SEM) and transmission electron microscopy (TEM). The third aliquot was fixed with 4% of paraformaldehyde in PBS and embedded in paraffin. Two 4-μm sections from all samples were stained with H&E.

### Scanning electron microscopy (SEM)

The fixed PO forms were then fixed in 1% osmium tetroxide followed by sequential dehydration steps from 25% to 100% ethanol. Ethanol was exchanged for liquid CO_2_ (Samdri Critical Pont Dryer). Using an inverted microscope, samples were positioned in stubs and gold coated (SPI-Module Sputter coater). Specimens were observed using a Zeiss 1450EP Scanning Electron Microscope.

### Transmission electron microscopy (TEM)

The fixed PO forms were then fixed in 1.5% osmium tetroxide, dehydrated in ethanol, and embedded in epoxy, and 50-nm sections were cut. Sections were then stained with lead hydroxide and uranyl acetate and were examined using a Phillips electron microscope (Phillips Electronic Instruments, Eindhoven, The Netherlands) operating at 75 kV.

### Infection of rats with PO forms

To determine the viability and infectivity of the PO forms, 15 day old Hotzman rats, purchased from Universidad Peruana Cayetano Heredia (UPCH), Lima, Peru, were infected intracranially (in the bregma) as described by Verastegui [16] with 15 and 30 day old PO forms. Rats were anaesthetized with ketamine (100 mg/kg body weight) and xylazine (5 mg/kg body weight) before infection. Eight rats were inoculated with ten 15 day PO forms in 100 μL of saline solution, nine rats were inoculated with five 30 day PO forms in 100 μL of saline solution, and five rats were inoculated with 100 μL of saline solution as control. The syringe needle gauge was 24G for 15 day PO forms and 21G for 30 day PO forms. PO forms of 60 days of development were not used for this experiment, as parasites of that age were 2.5 mm in diameter.

Necropsy was done four months later. Briefly, the rats were anaesthetized with ketamine (100 mg/kg body weight) and xylazine (5 mg/kg body weight). Anaesthetized rats were perfused with 200 ml of PBS and then with 100 ml of 4% paraformaldehyde in PBS. Brains were carefully removed, post-fixed for 24 hours at 4°C with 4% paraformaldehyde in PBS, and stored in 70% ethanol. Brains were observed macroscopically to identify extraparenchymal cysticerci; coronal sections of the brain (5 mm) were done until the intraparenchymal cysticerci were observed. Coronal sections of the brain were paraffin embedded, cut in 3 μm thick sections and Masson’s trichrome stained.

### Ethics statement

All animal procedures were performed in strict accordance with the recommendations in the Guide for Care and Use of Laboratory Animals of the National Institutes of Health. The protocol was approved by the Committee on the Ethics of Animal of the Universidad Peruana Cayetano Heredia, Lima, Peru (Permit Number: 61242).

### Western blot

The total proteins present in PO forms, activated oncospheres, and cysticerci were incubated with anti-oncosphere and anti-cysticercus rabbit sera in order to evaluate the change in the expression of antigenic proteins during PO form development. Total proteins of the PO form were obtained from PO forms cultured in HCT-8 cells as described previously. PO forms were collected at 15, 30, and 60 days of incubation, rinsed three times with PBS buffer, sonicated, and centrifuged. Hatched and activated oncospheres were obtained as described above; cysticerci removed from an infected pig were also rinsed, sonicated and centrifuged. The supernatants were used as total protein, and examined by western blot.

Protease inhibitors of 1mg/ml of Leupeptin (1/1000) (Sigma, St. Louis, Missouri), 0.1 M Pefabloc SC PLUS (1/100) (Roche, Mannheim, Germany) and 1mg/ml of Pepstatin (1/1000) (Sigma, St. Louis, Missouri) were added to the total protein prior to storing at -70°C. Three μg of total proteins were resolved in 15% SDS-PAGE non-denaturing gels and transferred to nitrocellulose (NC) membranes (Bio-Rad Laboratories, Hercules, CA). NC membranes were incubated overnight at 4°C with rabbit sera immunized with total proteins of activated oncosphere and cysticerci (1:200) [15]. The NC membranes were washed three times with PBS 0.3% Tween-20, incubated with peroxidase-conjugated goat anti-rabbit antibodies diluted 1: 400 (KPL, Gaithersburg, MD) for 1 hour at room temperature and resolved with DAB substrate (Sigma Chemical Co).

### Data analysis

The *in vitro* maturation assay using various cell lines was independently repeated three times and the development of PO form was expressed as percentages (%). The sizes of the oncospheres for each development time were measured and the result was expressed in micrometers (μm) as the mean size of oncosphere with 95% confidence intervals (CI). The Student's t-test was also performed to evaluate the difference between the sizes of the PO form cultured with and without transwell system, using the software Stata 10.0 with a significance level of 0.05.

## Results

### Development of PO forms

#### First experiment

Twelve cells lines were evaluated to determine the most suitable line for *in vitro* cultivation of *T*. *solium* activated oncospheres to obtain PO forms. The majority of cell monolayers remained confluent during the two week maturation period. After two weeks of culture, a high percentage (32%) of PO forms was obtained using HCT-8 cells in comparison to the other cell lines (Table 1). This cell line was therefore selected to study the morphology, protein expression, and antigenic response to total PO form proteins. Hutu- 80 and LLC-MK2 cell lines did not support PO form development.

**Table 1:** Development of *Taenia solium* postoncospheral forms at two week of incubation using different cells lines.

Cell line	Organism	Tissue	Postoncospheral form	
			Total of 10000	%
Hutu-80	Human	Duodenum	0	0
INT-407	Human	Embryonic intestinal	750	8
HCT-8	Human	Colon	3240	32
HT-29	Human	Colon	990	9
CaCo-2	Human	Colon	1500	15
SH-SY5Y	Human	Bone marrow	390	4
BeWo	Human	Placenta	630	6
MRC-5	Human	Lung	1320	13
H9c2	Rat	Heart	2520	25
CHO-K1	Hamster	Ovary	390	4
VERO	African Green Monkey	Kidney	1800	18
LLC-MK2	Rhesus Monkey	Kidney	0	0

#### Second experiment

In order to determine if the nutrients and secreted growth factors from HCT-8 monolayer feeder cells are necessary for the development of PO form, we cultured oncospheres in the absence of HCT-8 monolayer feeder cells. PO forms did not develop in either media alone, supernatants of HCT-8 monolayer cells, or supernatants of HCT-8 cells incubated with 2 week old PO forms.

#### Third experiment

In order to determine if oncospheres need direct contact with the cells to develop into PO forms, we co-cultured the cells and the oncospheres in a transwell system. Table 2 shows that the oncospheres develop to PO forms in both the transwell system, regardless of the type of membrane (collagen or polyester membrane) and in direct contact with the monolayer feeder cells. This experiment also shows that PO forms that grow in direct contact with feeder cells were larger (mean 176 μm) than PO forms that grow in a transwell system (112 μm and 76 μm for collagen coated and polyester, respectively) (p = 0.0006). Additionally, in the absence of feeder cells, there was not a significant difference in the size of PO forms among the transwell systems and plate (Table 2), we also observed that parasite started to die at two week of culture.

**Table 2:** *Taenia solium* postoncospheral forms size at two week of incubation using transwell system.

		With Monolayer feeder cell. Mean [Min-Max]	Without monolayer feeder cells. Mean [Min-Max]
**With transwell**	Collagen coated	112 μm [53 to 281 μm]	34 μm [20 to 69 μm]
	Polyester	76 μm [30 to 234 μm]	23 μm [15 to 32 μm]
**Without transwell**		176 μm [83 to 337 μm]*	21 μm [17 to 27 μm]

* P<0.05

### Morphology of PO form

**Fig 1:**
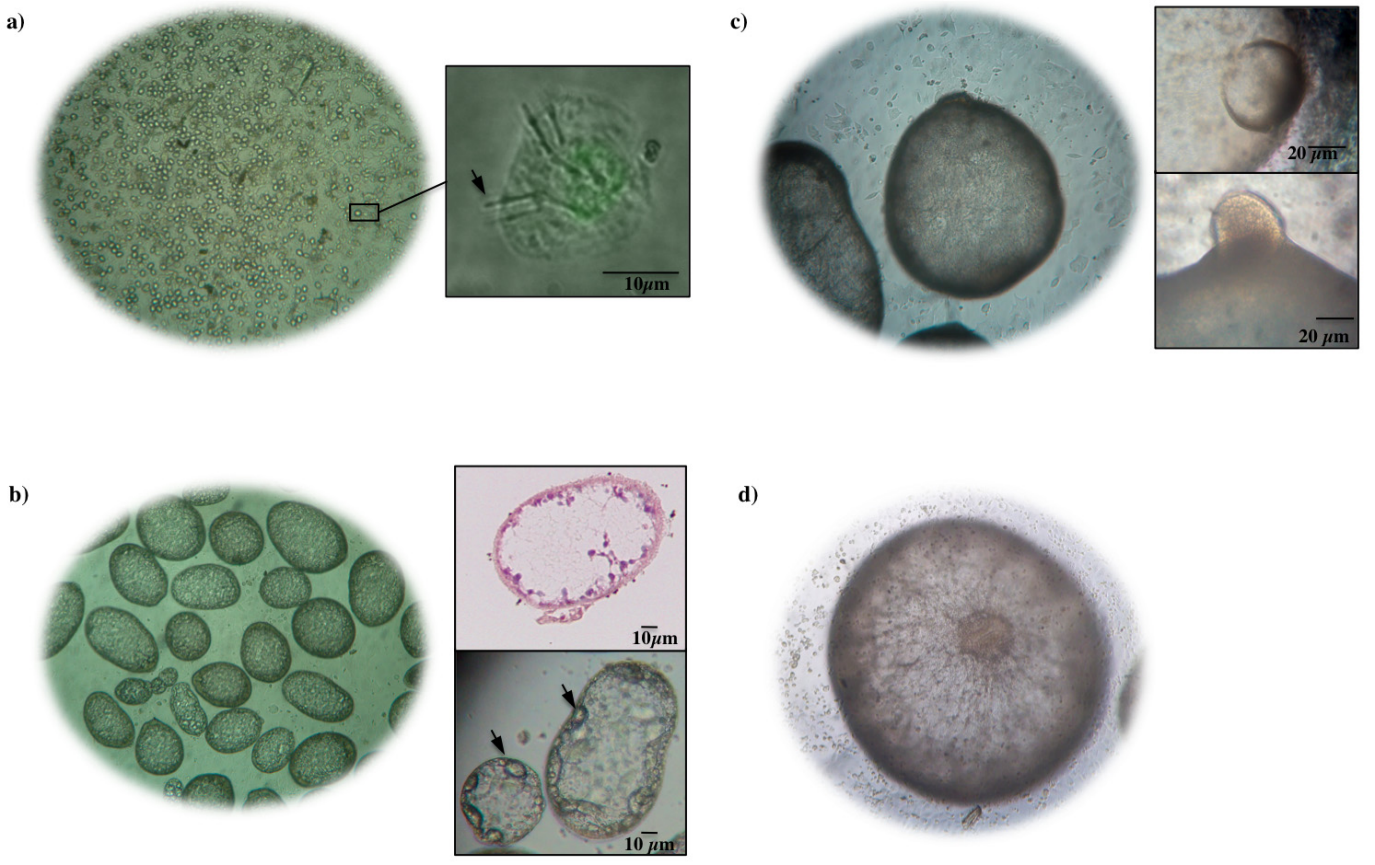
Light micrographs of development of *Taenia solium* postoncospheral form in HCT-8 cell monolayer. (a) Left: *T*. *solium* activated oncosphere at 1 day of culture viewed at 10x objective; Right: oncosphere with typical hooks. (b) Left: *T*. *solium* postoncospheral forms at 15 day of culture viewed at 10x objective; Right: nucleus and cells were present in small postoncospheral forms. (c) Left: *T*. *solium* postoncospheral forms at 30 days of culture viewed at 10x objective; Right: protuberance was observed in one end of the body. (d) *T*. *solium* postoncospheral forms at 60 days of culture viewed at 10x objective; structure like a belly button observed in the middle of the body.

Using inverted microscopy, we observed that at 1 day of culture in HCT-8 cells, the activated oncospheres did not change their size and form but were attached to the cell monolayers. At this time, the activated oncospheres measured from 18 μm to 25 μm in diameter (mean = 20.8 μm, 95% CI = 19.7–21.8 μm) with six hooks enclosed within the oncospheral tegument (Fig 1A).

At 3 days of culture in HCT-8 cells, the oncospheres changed their morphology and size, and we termed them postoncospheral (PO) forms. Between 3 and 12 days of culture the PO forms were spherical and larger in size (from 22 μm to 40 μm in diameter), the hooks were still visible and some PO forms remained attached to the cell monolayers. The oncospheres matured while attached to the cell monolayers for two weeks; after that they detached and became free-floating parasites. From 15 to 60 days of culture, the PO forms were not attached to cell monolayers, the hooks were not present and the size increased ten or more times in comparison to the initial size of the activated oncosphere.

The 15 day PO forms measured from 64 μm x 96 μm to 240 μm x 337 μm in size. There were 15 day PO forms with spherical and oval forms (Fig 1B), while some also had a protuberance. The six hooks were no longer present in the 15 day PO forms. The smallest 15 day PO forms (64 μm x 96 μm) presented cells inside of the tegument observed by H&E stain (Fig 1B). The 15 day PO forms showed motility that began with an oval shape, and then one polar ends of their body began to stretch, forming a protuberance and widening until it returns to its initial form (S1 Multimedia file).

The 30 day PO forms ranged in size from 840 μm x 980 μm to 1418 μm x 1617 μm, with an oval form. The formation of a protuberance was also observed in one of the polar ends of the body of 30 day PO forms while moving (Fig 1C and S2 Multimedia file).

The 60 day PO form reached up to 2500 μm in size (mean = 1991.6 μm and 95% CI = 1636.6–2346.6 μm) and had a spherical form like a cysticercus but without a scolex (S3 Multimedia file). Some of the parasites formed a structure like a “belly button” caused by cell accumulations in the middle of their bodies while they were moving (Fig 1D).

**Fig 2:**
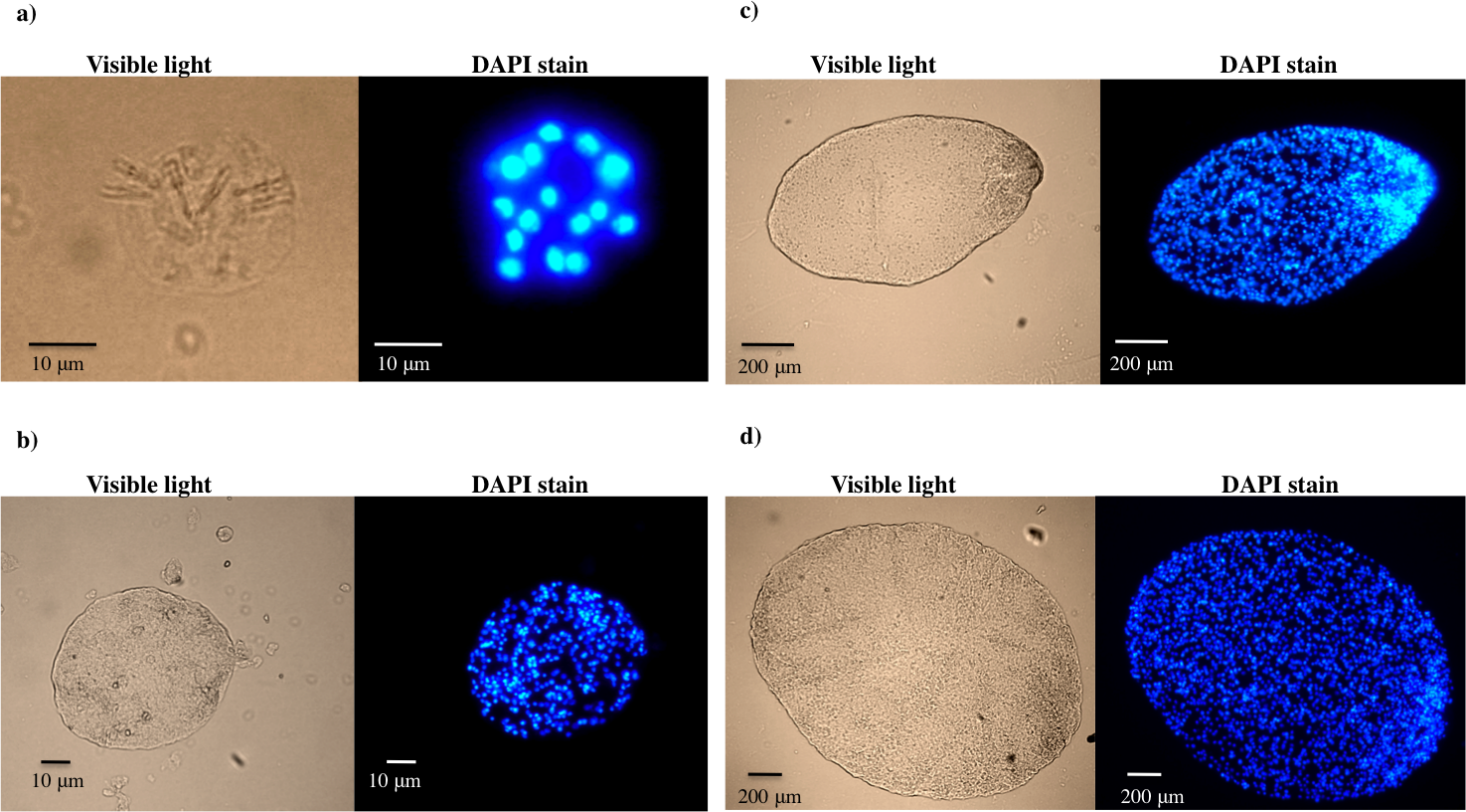
Fluorescence microscopy of *T*. *solium* postoncospheral forms stained with DAPI stain. Number of nuclei in *T*. *solium* activated oncosphere (a) compared with the number of nuclei in *T*. *solium* postoncospheral forms at 15 (b), 30 (c) and 60 (d) days of culture.

The number of cell nuclei within PO forms increased with age, as noted with the DAPI stain (Fig 2). Activated oncospheres had from 8 to 18 cell nuclei while PO forms had from 85 to 377 cell nuclei at 15 days of growth, between 1500 to 4000 cell nuclei at 30 days of growth, and more than 5000 cell nuclei at 60 days of growth.

Another important observation was that after 60 days of culture, the parasite showed many vesicles in its tegument; they began to degenerate, lost their ability to move, and finally died.

### Scanning electron microscopy (SEM) and Transmission electron microscopy (TEM)

**Fig 3:**
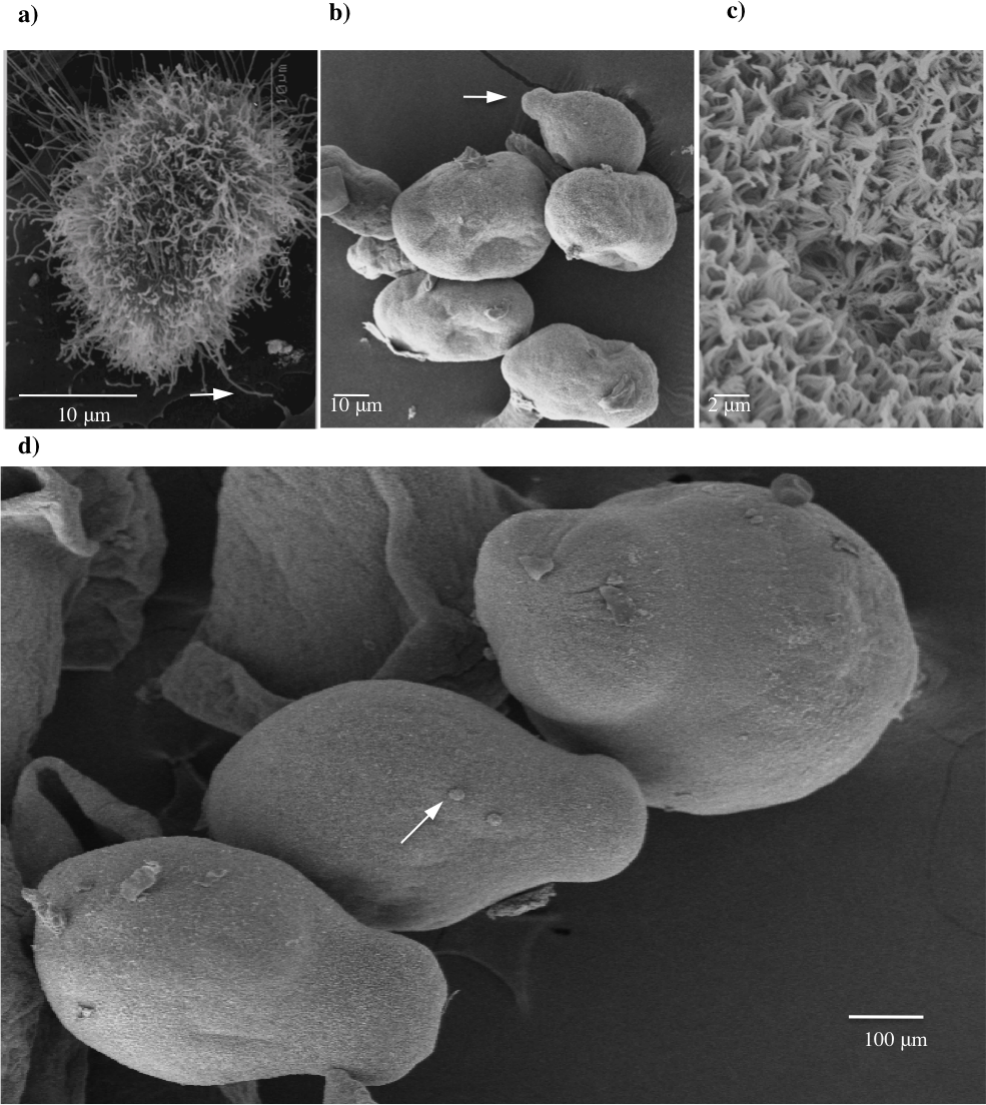
Scanning electron micrographs of *T*. *solium* postoncospheral forms. (a) *T*. *solium* activated oncosphere showing elongated microvilli. (b) 15 day postoncospheral forms showing the protuberance at one end of the body. (c) Close-up of the tegument of 30 day postoncospheral form showing microtriches. (d) 30 day postoncospheral form showing vesicles.

**Fig 4:**
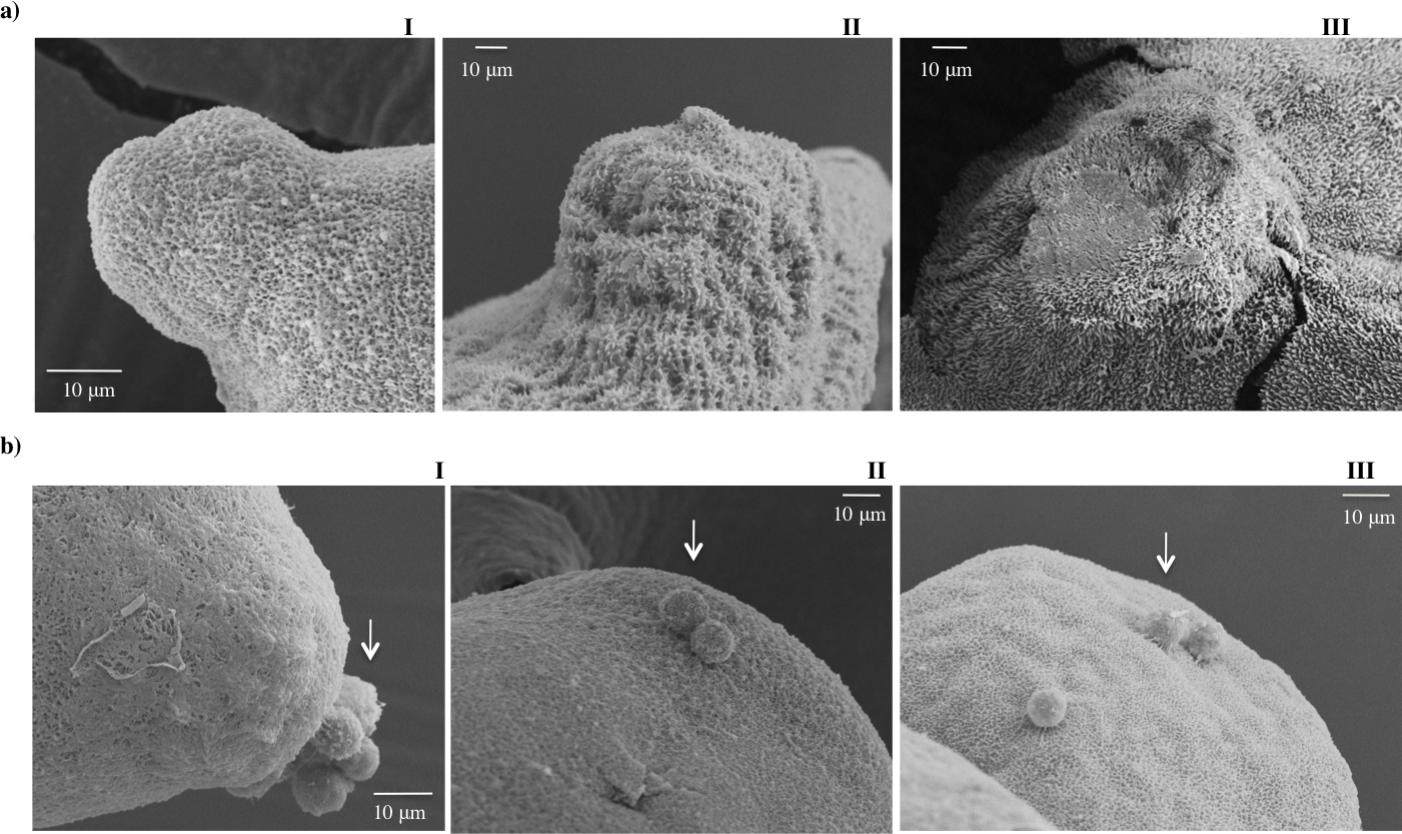
Scanning electron micrographs of the protuberance forming in *T*. *solium* postoncospheral forms at three growth points. (a) Microtriches and (b) vesicles (arrows) observed in the surface of the protuberance at one end of the body at 15 (I), 30 (II) and 60 (III) days PO forms.

In this study, we observed that *T*. *solium* oncospheres have microvilli on the surface (Fig 3A) while 15 day-PO forms to 60 day PO forms do not have microvilli (Fig 3B and 3D); instead, the PO form has structures like microtriches on the surface as observed in the Figs 3C and 4A.

They are long and fine and intertwine, forming something like a network (Fig 3C). Microtriches were homogeneously distributed across all the surfaces of the PO forms at all three growth times. The microtriches present in the protuberance of the 30 and 60 day PO forms were more developed than 15 day PO forms (Fig 4A).

Vesicles have also been observed in the PO forms at 15, 30, and 60 days. These vesicles were observed on the surface of the body and also in the protuberance of the PO form (Fig 3D and Fig 4B). In the protuberance, the number of vesicles was higher in 15 day PO forms than in 30 and 60 day PO forms (Fig 4B).

**Fig 5:**
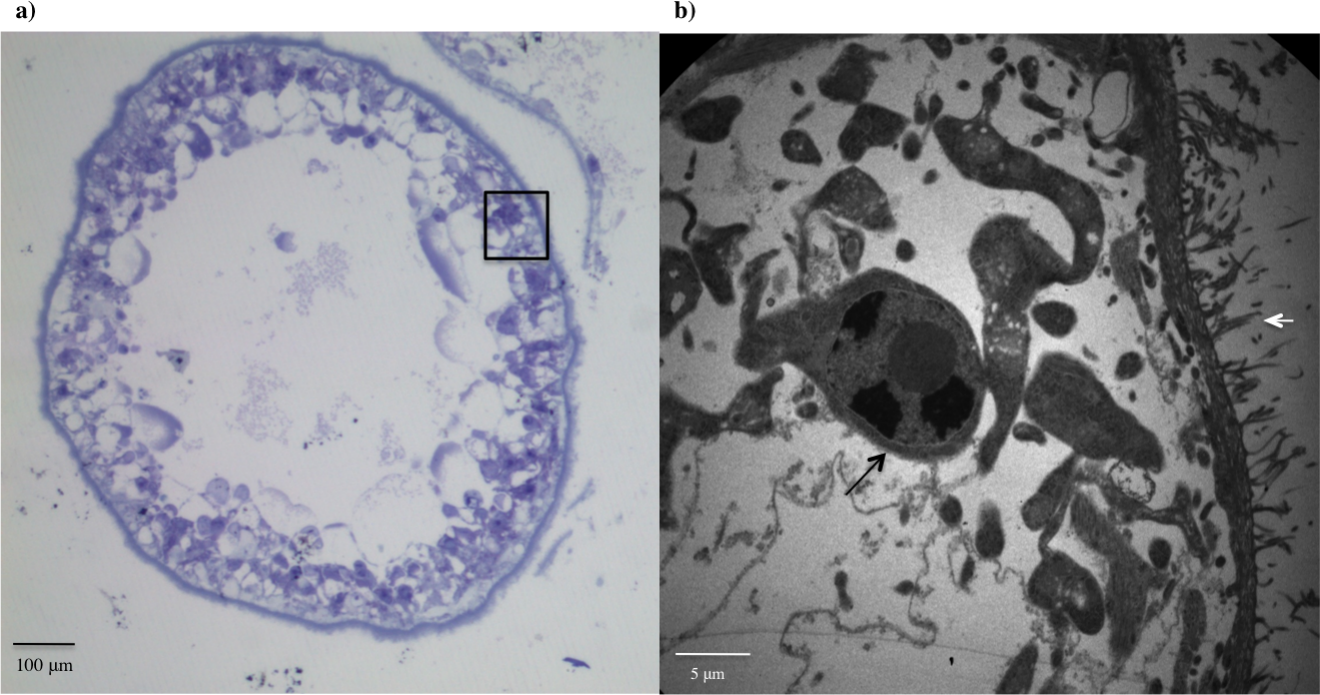
Morphology of *T*. *solium* 30 day postoncospheral form. (a) Haematoxylin stain of 30 day postoncospheral form showing a large central cavity and the tegument. (b) Transmission electron microscopy of the tegument of 30 day postoncospheral form showing microtriches and cells.

A large central cavity surrounded by a tegument was observed at 15, 30, and 60 days. The tegument was covered by microtriches; cells were clearly observed below the tegument in 30 day PO form as observed in Fig 5B.

### Development of PO forms to cysticerci

**Fig 6:**
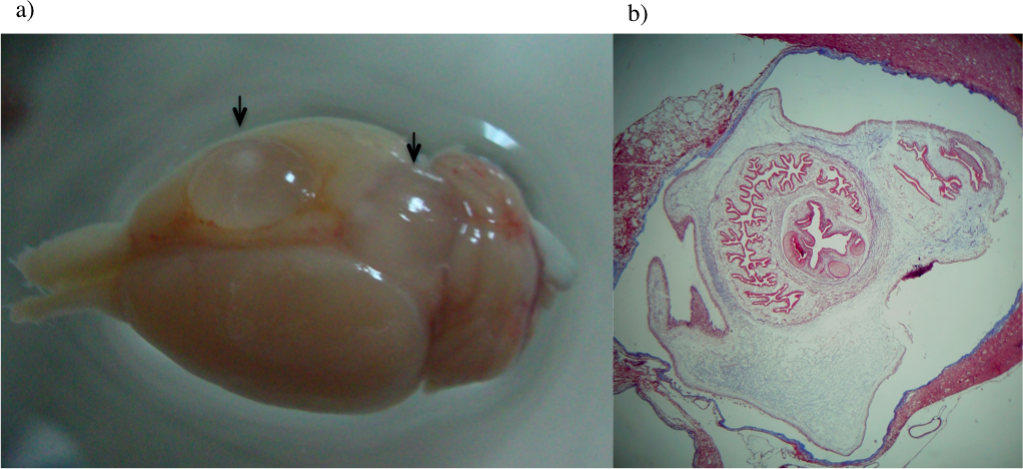
Rat brain infected with *T*. *solium* postoncospheral forms. (a) Rat brain 4 months post infection showing two extraparenchymal cysticerci (arrows). (b) Coronal sections of the brain containing an intraparenchymal cysticercus stained with Masson’s Trichrome staining showing tegument and scolex.

The 15 and 30 day PO forms developed to mature, viable cysticerci when rats were inoculated intracranially (Fig 6).

In rats infected in the brain with 15 day PO forms, 5/8 (63%) developed viable cysticerci with a scolex of which 4/5 (80%) developed more than 5 cysts in the brains examined. In rats infected with 30 day PO forms, 6/9 (66%) developed viable cysticerci of which 4/6 (77%) developed 5 cysticerci in the brain. These findings demonstrate that PO forms are viable and competent to become *T*. *solium* cysticerci with normal morphology.

### Comparison of antigenic proteins

In this study, we also evaluated if the PO forms changed the expression of antigenic proteins during development. The antigenic proteins were analyzed using rabbit sera against *T*. *solium* oncospheres and cysticerci.

**Fig 7:**
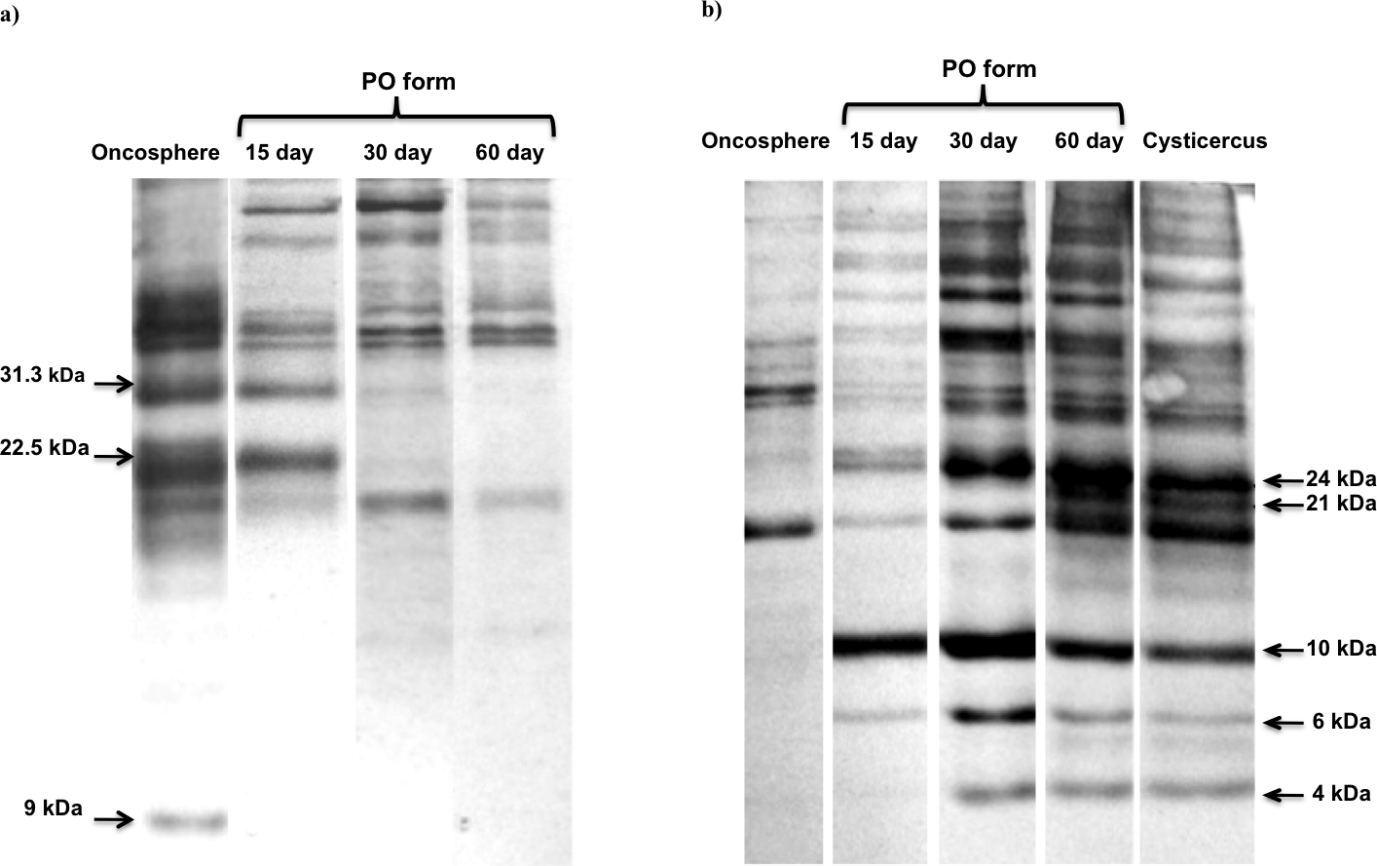
Western blot of *T*. *solium* antigens at different forms. (a) Using rabbit anti-oncosphere sera. (b) Using rabbit anti-cysticerci sera.

Anti-oncosphere antibodies demonstrated a band of 9 kDa expressed exclusively by the activated oncosphere. Some antigenic oncosphere proteins (22.5kDa and 31.3kDa) were only expressed by oncospheres and 15 day PO forms but not by 30 and 60 day PO forms (Fig 7A).

Using anti-cysticerci antibodies, we observed that PO forms also express some antigenic cysticerci proteins (24, 10, and 6 kDa), which were present at 15, 30, and 60 days. A 4 kDa antigenic band appeared only after 30 days of culture, and a 21 kDa band appeared only after 60 days of culture (Fig 7B).

## Discussion

This study shows that different cell lines are capable of supporting the *in vitro* development of *Taenia solium* postoncospheral (PO) forms. We described the development and morphological changes of the PO forms in HCT-8 cells at three time points of development (15, 30, and 60 days). The PO forms were able to develop into mature cysticerci when inoculated in rat brains. The pattern of PO form antigenic protein expression begins similarly to that of oncospheres, but it later becomes more similar to the expression of cysticerci antigenic proteins.

To our knowledge, this is the first study to achieve *in vitro* growth of *T*. *solium* PO forms. Two previous studies demonstrated that cell monolayers could be used to obtain the PO forms of *T*. *ovis* [9] and *T*. *saginata* [10], although it was unclear whether contact with the monolayers was necessary for the PO form development.

Our results show that oncospheres developed to PO forms in the presence of monolayer feeder cells. The PO forms that grew in direct contact with feeder cells were bigger than PO forms that grew in a transwell system with cells. It is likely that the parasite development depends on nutrients, growth factors, and cytokines secreted by feeder cells, and also the direct contact with cells might promote the uptake of nutrients through the parasite tegument. By contrast, the development of the parasite was incomplete in the absence of feeder cells, even when we added the supernatants of HCT-8 cells; it is probable that the secreted factors are very labile and need to be constantly produced to maintain the development of the parasite. The importance of secreted factors from feeder cells has been studied in the development of the *Echinococcus multilocularis* larval stage; they were cultured with rat hepatocytes which strongly stimulated vesicle regeneration, and suggested that soluble factors secreted by host cells acted on germinal cells as initiators of metacestode formation [17]. Similarly, PO forms of *Echinococcus granulosus*, *T*. *hidatigena*, *T*. *ovis*, *T*. *pisiformis*, *T*. *serialis*, *T*. *saginata* and *T*. *taeniaeformis* have been successfully obtained in the absence of feeder cells [11] [12]). There may be nutritional differences between parasite species or farming systems used *in vitro*. For instance, Heath et al used serum from each specific intermediate host in the culture medium while in the present study we used fetal bovine serum. Serum contains specific factors for each host-parasite system that stimulate development of the parasite [11].

Interestingly, the PO forms did not grow in LL-CMK2 monolayer cells which may be due to lack of essential nutrients, growth factors, cytokines or other possible growth stimuli, and the fact that different cell lines likely express different surface molecules. The transformed cell lines that are used in the present study usually express a variety of growth factors that normal cells would not produce in their tissues of origin. These factors may also explain the different growth rate of the parasites among the cell lines.

We chose to study the PO forms from day 15 forward, because it was at this point that the parasite detached from the cell monolayers and became free floating. We hypothesized that the loss of ability of PO form to attach to the cell monolayers is due to loss of the microvilli that are present only in activated oncospheres [15]. We observed structures that look like microtriches in 15, 30, and 60 day PO forms. The change from microvilli to microtriches has been reported in *Echinococcus granulosus* [18] and a similar process likely happens in the PO form of *T*. *solium*. However, we do not know if the microvilli turn into microtriches or if they are lost and replaced by microtriches. Chervy et al., 2009 mention that the microtriches are either formed *de novo*, or via the conversion of microvilli by the addition of electron-dense material to form the cap [19].

Another characteristic of 15, 30, and 60 day PO forms was the absence of hooks. Our results are similar to those found *in vivo* in which the 6 hooks were lost 10–15 days after experimental infection of pigs [13]. The hooks play a major role in the oncosphere activation, by shedding the oncospheral membrane [20]. However it is not clear if hooks have another role during the infection process such as penetrating the intestine.

Vesicles were present in the PO forms at all three time points, with more vesicles at the surface of the anterior pole in the 15 day PO forms. Previous studies have demonstrated that oncosphere vesicles are important for attachment to host tissues and to facilitate penetration through the epithelium or for protection against digestive enzymes and evasion of the immune response [21], [22]. However, vesicles of the PO form may contribute to evasion of the immune response and in the formation of the tegument as it has been shown for *Echinococcus granulosus* [22].

The *T*. *solium* PO form has the general pattern of postoncosphere development reported in *Taenia serialis*, *Taenia ovis*, *Echinoccoccus granulosus*, *Taenia hydatigena* and *Taenia pisiformes* when these are grown in medium supplemented with serum [11]. In our study, the pattern observed in *T*. *solium* postoncospheral development was 1) cell multiplication as seen with DAPI stain, 2) cavity formation, which was observed from 15 to 60 days PO forms, and 3) further growth, and where applicable movement which is observed in all three age points. Additionally, when the 15 and 30 day PO forms were injected into rat brains they continued to mature and formed cysticerci, proving their viability; this is a highly efficient method of infecting an animal model. Finally, the PO forms started to die off at 60 days of culture.

No scolex formation was observed in this *in vitro* study. First, it is likely that the PO forms were not viable long enough to complete its development as it was reported for *T*. *saginata* [10]. Secondly, it is possible that the development was stunted because specific host signals or nutrients present in the serum or tissue from their intermediate host were missing as reported in other species of taeniid [11]. However, the protuberance that we observed at one end of the PO form and the “belly button” structure described in the center of the parasite at 60 day old could be the initial development of the scolex region, as described for other taeniids [11].

We observed the transition of immunogenic proteins from oncosphere to cysticerci. The 15 day PO forms stopped producing some oncosphere proteins, such as those of the size of 22.5 and 31.3 kDa, and, as the PO forms aged, started the expression of a majority of the cysticerci antigenic proteins. Bands of 22.5 and 31.3 kDa were reported as specific *T*. *solium* oncosphere antigens [23]; however, in this study, we observed that these antigenic proteins also were expressed in 15 day PO forms, indicating at least some temporal overlap. The changes in antigenic protein expression during the development of PO forms may help with evasion of the host immune system. In other taeniid species, these changes are associated with the formation of the tegument, and with changes in the parasite’s susceptibility to antibody and complement-mediated attack early in the development of the metacestode [24]. The changing expression of antigenic proteins over the course of the PO forms could be used when designing diagnostic assays, and help determine if a particular protein is a good vaccine candidate.

In conclusion, we have developed and standardized a method for culturing *T*. *solium* oncospheres to obtain PO forms for up to 60 days in the presence of an HCT-8 feeder cell monolayer. These PO forms can complete their development to mature cysticerci when injected in rat brains, and changes in the expression of antigenic proteins during their development are related to the changes observed in their morphology.

## Supporting Information

S1 Multimedia File Motility of 15 day *T*. *solium* postoncospheral form. (MOV)

S2 Multimedia File Motility of 30 day *T*. *solium* postoncospheral form showing the formation of the protuberance in one of the polar ends of the body. (MOV)

S3 Multimedia File Motility of 60 day *T*. *solium* postoncospheral form. (MOV)
